# Bioactive Compounds and Antioxidant Activity in Different Types of Berries

**DOI:** 10.3390/ijms161024673

**Published:** 2015-10-16

**Authors:** Sona Skrovankova, Daniela Sumczynski, Jiri Mlcek, Tunde Jurikova, Jiri Sochor

**Affiliations:** 1 Department of Food Analysis and Chemistry, Faculty of Technology, Tomas Bata University in Zlin, nam. T.G. Masaryka 5555, CZ-760 01 Zlin, Czech Republic; E-Mails: sumczynski@ft.utb.cz (D.S.); mlcek@ft.utb.cz (J.M.); 2 Institut for Teacher Training, Faculty of Central European Studies, Constantine the Philosopher University in Nitra, Drazovska 4, Nitra SK-949 74, Slovakia; E-Mail: tjurikova@ukf.sk; 3 Department of Viticulture and Enology, Faculty of Horticulture, Mendel University in Brno, Valticka 337, CZ-691 44 Lednice, Czech Republic; E-Mail: sochor.jirik@seznam.cz

**Keywords:** berry, bioactive compounds, antioxidant activity, phenolic compounds, anthocyanins, health benefits

## Abstract

Berries, especially members of several families, such as Rosaceae (strawberry, raspberry, blackberry), and Ericaceae (blueberry, cranberry), belong to the best dietary sources of bioactive compounds (BAC). They have delicious taste and flavor, have economic importance, and because of the antioxidant properties of BAC, they are of great interest also for nutritionists and food technologists due to the opportunity to use BAC as functional foods ingredients. The bioactive compounds in berries contain mainly phenolic compounds (phenolic acids, flavonoids, such as anthocyanins and flavonols, and tannins) and ascorbic acid. These compounds, either individually or combined, are responsible for various health benefits of berries, such as prevention of inflammation disorders, cardiovascular diseases, or protective effects to lower the risk of various cancers. In this review bioactive compounds of commonly consumed berries are described, as well as the factors influencing their antioxidant capacity and their health benefits.

## 1. Introduction

In the last few decades there has been a constant increase of popularity and an interest regarding research of all kinds of fruits. Particularly fruit berries are well studied, as they contain the best dietary sources of bioactive compounds (BAC) [1,2,3,4]. They are abundant especially in highly-colored berries. To the species that contain the most BAC belong members of several families, such as Rosaceae (strawberry, raspberry, blackberry), and Ericaceae (blueberry, cranberry). They are globally known and consumed, and berries’ BAC are used as functional food ingredients. Additionally, grape berries (genus *Vitis*) and their products (juice, wine) are great sources of BAC [5,6,7,8,9]. To the berry group belong other relevant types of berries with low to medium BAC content, but less noted or applied as nutraceuticals, such as bilberries (*Vaccinuim myrtillus*) [10,11], elderberries (*Sambucus* spp.) [12,13], gooseberies (*Ribes uva-crispa*) [14], cape gooseberies (*Physalis peruviana*) [15], chokecheries (*Prunus virginiana*) [16], arctic brambles (*Rubus articus*) [17], cloudberries (*Rubus chamaemorus*) [18], crowberries (*Empetrum nigrum*, *E. hermaphroditum*) [19], lingonberries (*Vaccinium vitis-idaea*) [20], loganberies (*Rubus loganobaccus*), marionberries (*Rubus* spp.) [21], honeyberies (*Lonicera caeulea*) [22], Saskatoon berries (*Amelanchier alnifolia*) [23], Rowan berries (*Sorbus* spp.) [24], maqui [25], and sea buckthorn (*Hippophae rhamnoides*) [26].

Berries, fruits full of BAC, are also very delicious, have low energy, and are often consumed in fresh form when the most BAC are still active and in the greatest amount. To the BAC group in berries belong antioxidants such as phenolic compounds and fruit colorants (anthocyanins and carotenoids). Berries’ phenolics represent a diverse group of compounds including phenolic acids, such as hydroxybenzoic and hydroxycinnamic acid conjugates; flavonoids, such as flavonols, flavanols, and anthocyanins. In addition, tannins, divided into condensed tannins (proanthocyanidins) and hydrolyzable tannins, are reported to be important BAC. To bioactive compounds belong other antioxidants such as vitamins (ascorbic acid) and minerals with antioxidant properties. These compounds are of great interest for nutritionists and food technologists due to the opportunity to use BAC as functional foods ingredients. Nutraceuticals and functional foods have become very popular for people due to the consumer demands for healthy nutraceutical foods that could possibly reduce some health risks and improve various health conditions. Due to the market for functional foods in the EU having grown, the years 1999 to 2006 saw the market increase from about $1.8 billion to $8 billion [27].

Many BAC, individually or combined, possess high antioxidant capacity. Antioxidants are the substances that can scavenge free radicals. These radical forms have an unpaired electron in the outer orbit that results in their instability and reactiveness. The human body possesses defense mechanisms against free radical-induced damage, such as “oxidative stress”, but cumulative oxidative damage leads to various diseases. Additionally, some dietary antioxidants may help to decrease the incidence of oxidative stress-induced damage. It is supposed that there is an association between antioxidant-rich diets and the reduction of oxidative damage to DNA. Therefore, antioxidants could be a prevention of some crucial points in carcinoma genesis [28,29]. However, the effective physiological relevance of foods with antioxidant intake is uncertain as many investigations are mainly based on *in vitro* assays. Therefore, their findings do not necessarily correspond with human physiological mechanisms *in vivo* [30]. Therefore, the effects of dietary antioxidants *in vivo* should be studied intensively to know their physiological effects.

This review will be aimed mainly at BAC and antioxidant capacity of globally known, commonly-consumed fruit berries with the highest content of BAC, such as strawberries, blackberries, raspberries, blueberries, and cranberries. Berries are a profitable source of BAC, and both phenolics and ascorbate. Considering that berry fruits are often consumed in fresh form, their antioxidant capacity is not reduced due to any contrarious influences during processing, such as heat or oxidation [31]. Berry phenolics are transformed by the human metabolism and by colonic microflora into related molecules that can persist *in vivo* and gather in target tissues. There, they can promote the abundant biological effects of berries [32].

### Chemical Composition of Berries and BAC

The chemical composition of represented berries is variable depending on the cultivar and variety, growing location and environmental conditions, plant nutrition, ripeness stage, and time of harvest, as well as subsequent storage conditions. Therefore, the content of each individual component and the quality of the fruits is highly variable.

Berries, in general, are rich in sugars (glucose, fructose), but low in calories. They contain only small amounts of fat, but a high content of dietary fiber (cellulose, hemicellulose, pectin); organic acids, such as citric acid, malic acid, tartaric, oxalic and fumaric acid; certain minerals in trace amounts (*i.e.*, 100 g of edible portion of raspberries, blackberries, or blueberries could provide more than 50% of Recommended Dietary Allowance (RDA) for manganese [33,34]); some vitamins (ascorbic acid and folic acid); and phytochemicals, such as phenolic compounds. These compounds could be a good option for the food industry to use as functional foods ingredients.

Phenolic compounds belong to a wide and heterogeneous group of chemical components that possess one or more aromatic rings with a conjugated aromatic system and one or more hydroxyl groups. They tend to donate an electron or a hydrogen atom to a free radical and convert it into an inoffensive molecule. Therefore, phenolics have relevant *in vitro* and *in vivo* antioxidant activities. Phenolic compounds occur in free and conjugated forms with sugars, acids, and other biomolecules as water-soluble (phenolic acids, flavonoids and quinones) or water-insoluble compounds (condensed tannins).

To the relevant BAC in berries belong phenolic compounds that include flavonoids, such as anthocyanins (*i.e.*, cyanidin glucosides and pelargonidin glucosides), flavonols (quercetin, kaempferol, myricetin), flavanols (catechins and epicatechin). Furthermore, phenolic acids (hydroxybenzoic acids and hydroxycinnamic acids) and hydrolysable tannins, such as ellagitannins, act as important BAC. These components, either individually or combined, are mainly responsible for berry health benefits and are also associated with their antioxidant properties.

In addition to these components, ascorbic acid could be a very potent antioxidant occurring in significant amounts in fresh berries. Ascorbic acid is an essential water-soluble vitamin with excellent reducing properties, well known by its high antioxidant activity due to the neutralization of free radicals and other reactive oxygen species, formed via cell metabolism, which are associated with several forms of tissue damage and diseases. It is also considered as the nutrient quality indicator during processing and storage as it is known that if ascorbic acid is well-retained, the other nutrients could stay in foods with minimum changes and losses, too. The loss in ascorbic acid content is also cultivar-dependent [35]. However, this vitamin is a great reducing agent with high antioxidant activity [36]; in many studies it was evaluated to contribute only a small amount (up to 10%) to the total antioxidant capacity of the fruits [37,38,39,40].

## 2. Strawberries

Strawberries (family: Rosaceae, genus *Fragaria*, cultivated: *F*. × *ananassa*, wild: *F. virginiana*) belong to berries that are popular due to their desirable sweet taste and attractive aroma, with smooth texture and red color. The plant is acclimatized to different environments and, therefore, could be cultivated worldwide, intensively in Europe and North America in open fields, whereas in China it is cultivated mainly in greenhouses [41]. There were more than 600,000 acres and 3.9 million tons of strawberries produced worldwide in 2005. The area of more than half of that acreage was utilized for a strawberry farming in Europe. The next largest production regions for strawberries are the Russian Federation, and USA, which produced 1.1 million tons of strawberries [42].

Amongst the fruits, fresh strawberries are considered to be one with the highest content of ascorbic acid. Among the berry species, strawberries have similar content to raspberries, but about four-times more ascorbate than blueberries. Ascorbate content in strawberries is highly variable, and in fresh strawberries generally ranges from 5 to 50 mg/100 g fresh weight (fw) [37,43,44,45], in some cultivars up to 80 mg/100 g fw [46]. As it is known, there is a gradual decrease in ascorbate content as the storage temperature or duration increases. However, during a week of storage it was determined no ascorbate losses occurred in strawberries at various temperatures [37]. In contrast to that, the loss of ascorbic acid in fresh fruit juices increases with storage time, especially if the temperatures of storage are higher than the refrigerated conditions [45,46].

Strawberries have been referred in many sources of folk medicine and official pharmacopoeia as a potential remedy, *i.e.*, due to their astringent and diuretic properties [47]. In the form of fruit paste they are used in folk medicine to heal skin diseases and wounds, and the juice for inflammation of the nerves and lungs [48]. The leaf extract of strawberries has anti-diabetic, antioxidant, anti-inflammatory, and anti-apoptosis effects [49,50,51,52,53]. Antioxidants in strawberries also help to lessen the risk of cardiovascular incidents by inhibition of LDL-cholesterol oxidation, or improved vascular endothelial function. This could reduce the risk of incidence of thrombosis [54,55]. It is known that some compounds present in strawberries, such as ellagic acid and quercetin [53,56,57,58], have demonstrated anti-cancer activity in their purified forms or fractions, sometimes enriched with specific components. Crude extracts of strawberries and pure compounds of anthocyanins (cyanidin-3-glucoside, pelargonidin, and pelargonidin-3-rutinoside) show antioxidant and human tumor cell anti-proliferative activities *in vitro*. Thus, they could suppress the growth of human oral, colon, and prostate cancer cells [59,60]. The preventative effect of berry fruits for human esophageal cancer is because of their potential to modify exposure of several genes relating to the progress of oral cancer [61]. The protection from tumorigenesis upon pre-treatment with strawberry extracts was observed for breast cancer in mice [62], too, but the mechanism by which it exerts the chemoprevention is still not clear. Protective effects of strawberry extracts on human dermal fibroblasts was also referred [63,64].

### 2.1. BAC in Strawberries

The relevant BAC in strawberries are presented in Table 1.

**Table 1:** Phenolic composition and factors influencing the composition of strawberries.

Berry	Major Phenolic Compounds	Factors	References
Strawberry	*Phenolic compounds*		
		Cultivar, genotype, variety	[65,66,67,68,69,70,71,72,73]
		Growing location	[65]
		Cultivation techniques (conventional, organic)	[69,74,75,76]
		Cultivation condition (greenhouse, plastic tunnel, open-field, light)	[66,72,77,78,79]
		Growing season, ripening	[66,70,73,78]
		Processing	[80,81,82,83]
		Storage (time, temperature)	[69,83]
	*Flavonols*		[63,66,80,84,85]
	Kaempferol glycosides (Kaempferol-3-glucoside, Kaempferol-glucuronide, Kaempferol-3-malonylglucoside, Kaempferol-coumaroyl-glucoside) Quercetin glycosides (Quercetin-3-glucuronide, Quercetin-3-malonylglucoside, Quercetin-3-rutinoside = rutin, Quercetin-3-glucoside)		
	*Anthocyanins*		[63,68,69,71,84,85,86,87,88]
	Cyanidin glycosides (Cyanidin-3-glucoside, Cyanidin-3-rutinoside, Cyanidin-3-galactoside, Cyanidin-3-malonylglucoside) Pelargonidin glycosides (Pelargonidin-3-glucoside, Pelargonidin-3-rutinoside, Pelargonidin-3-galactoside, Pelargonidin-3-arabinoside, Pelargonidin-3-malonylglucoside, Pelargonidin-3-malylglucoside) Peonidin glycosides (Peonidin-3-glucoside)		
	*Phenolic acids and Hydrolyzable tannins*		[63,71,83,84,85,89]
	Ellagic acid and its glycosides		
	Ellagitannins		
	Gallic acid		
	Gallotannins		
	Caffeic acid		
	*p*-coumaric acid and coumaroyl glycosides		

The variability and an exact content of particular phenolic compounds of strawberries depend on many factors, such as genetic qualities, cultivation conditions, ripeness stage, storage time and conditions [65,66,67,68,69,70,71,72,73,74,75,76,77,78,79]. The content of the polyphenols such as cyanidin, pelargonidin, and quercetin glycosides displays are much more tightly regulated, supposing a consequent genetic control [90]. The identification of food phenolics is essential as their nature, size, solubility, degree, and position of glycosylation and conjugation, has an impact on their absorption, distribution, and metabolism in humans [84].

The total phenolic content (TPC) of strawberries is approximate to values in raspberries and blackberries, with variances due to the mentioned factors, but lower than in highbush and lowbush blueberries [37,91]. The phenol content in strawberries decreases during fruit development from the unripe to the ripened stage. A significant decrease (nearly 89%) was observed in ripened fruits as compared to green fruits. To compare conventional cultivation techniques to organic cultivation, no coherent effects on phenolics abundance in strawberries [74,75,76] was found.

Strawberries are usually eaten in fresh form but they are highly perishable with rapid deterioration in quality due to rapid spoilage. Due to that fact, the relevant part of the strawberry production is processed. Strawberries are consumed in processed products such as jams, jellies, puree, either as canned fruit, or syrup, in drinks as juices, *etc.* Freshly-produced strawberry juices have higher TPC, anthocyanin, and proanthocyanidin content, than those stored for six months at 4 and 30 °C [81]. The processing of the clear juice showed extensive losses of all phenolics.

Anthocyanins in strawberries are the major known polyphenolic compounds, responsible for fruit color, and can be used as natural pigments (red and blue colors) for the food industry. The anthocyanin content of strawberries, compared to other common berries, is much lower than in blueberries and blackberries, and lower than in raspberries [37,91]. Their occurrence is influenced by cultivar selection [68], environmental factors such as light, temperature, and agricultural methods. The degradation rate of anthocyanins is also time- and temperature-dependent [37]. An amount of strawberry anthocyanins rose to an average of 4.3-fold after a week of storage. The magnitude of that rise was attributed to temperature. After storage at 0 °C, anthocyanin content rose 1.7-fold, while at 30 °C storage, the obvious rise was 6.8-fold. Strawberry products, such as puree prepared under nitrogen or carbon dioxide, result in greater retention of anthocyanins than ones prepared under air. Thus, strict oxygen exclusion during strawberry processing appears to be convenient to improve anthocyanin stability, but some losses can occur under anaerobic conditions during storage [83]. Another factor affecting color and anthocyanin structure is pH. The pH modification can influence chemical reactions in phenolic compounds, such as anthocyanins. Lower pH (2.5) is better for the preservation of polyphenols in strawberry products during storage of a few months than higher values, as total anthocyanin content is correlated with antioxidant activity [92].

### 2.2. Antioxidant Capacity of Strawberries

The antioxidant capacity of berries, such as strawberries, is strongly related to the present effective oxygen radical scavengers. To those compounds belong phenolics, most of which express relevant *in vitro* and *in vivo* antioxidant activities and ascorbic acid. Considerable increases in the plasma total antioxidant capacity (TAC) and ascorbic acid during 16-day strawberry consumption (500 g of strawberries) were progressively observed after strawberry supplementation [93]. Plasma polyphenols, such as anthocyanins, after consuming strawberry beverages was also studied and pelargonidin-*O*-glucuronide was found as the most abundant metabolite. Higher concentrations of key strawberry compounds and metabolites are achieved with eating more strawberries [94].

Total antioxidant capacity (TAC) of strawberries is influenced by several factors (Table 1).

In general, the TAC values of strawberries are similar to raspberries and blackberries and less than in blueberry species. However, TAC of strawberries could be influenced by storage time and temperature that is accompanied by increases in strawberry anthocyanins [37]. The antioxidant capacity of strawberry fruit during period of a week could increase by an average of 1.5-fold, with the highest increase occurring at 10 and 20 °C than TAC of those stored at lower temperatures (0 or 5 °C) [69]. TAC also increases with maturation from green to red fruit [67].

The TAC could be influenced by silvicultural treatment such as organic cultural systems. Organically-grown strawberries exhibited generally higher activities in antioxidant enzymes, antioxidant capacity, and higher levels of antioxidants, such as flavonoid content [69]. The antioxidant capacity decreases with proceeding processing [81], except heat processing, which partly causes a growth due to the formation of products that are effective as antioxidants. Pressing and pasteurization are the most problematic processes for the degradation of BAC [82].

## 3. Red Raspberries

Red raspberries (family: Rosaceae, genus *Rubus*, common cultivated variety: *R. idaeus*) belong to the red-colored *Rubus* fruit cultivars grown in Europe (European red raspberry), North America (American variety), and many different cultivars and varieties in Asia, *i.e.*, *R. hirsute*’s growing in China [95]. Red raspberries are the fourth most significant fruit product in the world. The similarly planted areas of red raspberries include Europe and Asia. In 2005 Europe (Serbia and Montenegro, Poland) produced 231,000 tons, Asia (Russian Federation, mainly), 131,000 tons, while North America produced about 16% of the red raspberry tonnage in the world, in particular in the USA [42].

Raspberries are called bramble fruit and are an aggregate of drupelets. They have a very popular attractive flavor (taste and aroma) for consumers. They are also great source of vitamins such as ascorbic acid. Its content in fresh raspberries generally ranges from 5 to 40 mg/100 g fw. Among the berry species, raspberries have similar content to strawberries and blackberries, about three-times more ascorbate than blueberries have, but less than in red currants, and several times less than black currant vitamin content [37,96,97,98,99]. The content losses of ascorbate in raspberries (storage temperature or duration), by 44% after a week of storage, did not influence the antioxidant capacity of fruits [37].

The fruits have been used in traditional and alternative medicine for a long time to cure wounds, colic, diarrhea, and renal illnesses [100]. Raspberries could also be helpful in the diet targeted for managing early stages of type II diabetes and hypertension [101]. Raspberry extracts, some individual polyphenols (anthocyanins, ellagitannins, and ellagic acid) [102] or together with other compounds (*i.e.*, ascorbic acid, carotenoids) for synergetic effects, could inhibit proliferation of cancer cells *in vitro*. Raspberry extracts have shown anti-proliferative effects to suppress the growth of human colon, prostate, breast, and oral tumor cells [103,104,105,106,107] and the effect is comparable with other common berry extracts.

### 3.1. BAC in Raspberries

The relevant BAC in raspberries are presented in Table 2.

**Table 2:** Phenolic composition and factors influencing the composition of red raspberries.

Berry	Major Phenolic Compounds	Factors	References
Red Raspberry	*Phenolic compounds*		
		Cultivar, genotype, variety	[96,97,99,101,108,109,110,111,112,113,114,115,116]
		Growing location	[97]
		Cultivation techniques (conventional, organic)	[117]
		Cultivation condition (light, maturation)	[118]
		Growing season	[115]
		Processing (jam processing)	[119]
		Storage (time, material, atmosphere)	[99,108,120,121]
	*Flavonols and Flavons*		[109,110,113,117,122,123,124,125]
	Kaempferol glycosides (Kaempferol-glucuronide, Kaempferol-hexoside)		
	Quercetin glycosides (Quercetin-3-glucuronide, Quercetin-3-rutinoside = rutin, Quercetin-3-hexoside, Quercetin-3-rhamnoside, Quercetin-3-glucoside)		
	apigenin		
	chrysin		
	naringenin		
	*Anthocyanins*		[112,113,117,123,124,125]
	Cyanidin glycosides (Cyanidin-3-glucoside, Cyanidin-3-rutinoside, Cyanidin-3-sophoroside)		
	Pelargonidin glycosides (Pelargonidin-3-glucoside, Pelargonidin-3-rutinoside)		
	*Phenolic acids and Hydrolyzable tannins*		[99,109,110,113,122,123,125]
	Ellagic acid and its glycosides		
	Ellagitannins (sanguiin H-6, lambertianin C)		
	Caffeic acid		
	*p*-coumaric acid and coumaroyl glycosides		

The total phenolic content of raspberries, among the berry species, is approximately the same as in strawberries, about half of the phenolics amount than in blackberries and about four-fold less content than in blueberries. Red currants and black currants have phenolic content of around 2.5–3 times higher than in raspberries [37,96]. Raspberries, comparing with other berries, are most influenced by storage. Changes due to storage time and temperature could be examined in all their varieties. However, raspberry phenolics increased by about 1.5-fold after week storage. Additionaly, the content of raspberry anthocyanins could increase by about 2.5-fold after a week of storage at 20 °C. Changes are minor after 10 and 30 °C storage and minimal at 0 °C. It is followed by an almost two-fold increase in antioxidant capacity [37].

Raspberries could be consumed fresh, but due to their short storage life, are limited by rot and loss of firmness [108]. More often they are utilized as processed products, such as jams, jellies, purees and juices, ice creams or used as ingredients or for flavoring of various food products (yoghurts, smoothies). The freezing process affects the values of TPC only slightly [99].

The composition of the raspberry predominant anthocyanins (Table 2) can be used to differentiate red *Rubus* species from each other by reason that cyanidin in cultivated red raspberry is typically glycosylated with 3-sophorose (56%). In wild red raspberry there is about 30% of this form, and cyaniding-3-glucose content is about 27% [113]. As for the anthocyanin amounts among the berry species, raspberries have similar content to red currants, little more than strawberries, but about 2.5-times fewer anthocyanins than blackberries and about six-times fewer than black currants. The anthocyanin content of the three berry species varied more than 25-fold with blueberry > raspberry > strawberry [37,96].

### 3.2. Antioxidant Capacity of Raspberries

The total antioxidant capacity of the raspberries species and strawberries are similar to each other, but it is about three-fold lower than in blueberries. During their storage there could be an increase of TAC at temperatures > 0 °C (followed by the rise of anthocyanins content and TPC) [37].

Antioxidant capacity of raspberries is influenced by several factors as listed in Table 2. Amounts of antioxidants in berries could be affected by pre-harvest climate conditions, such as light intensity, day length, and temperature [121]. During the ripe stage there exists a linear connection between TAC values and an anthocyanin amount and TPC [70,109], organic culture [117], and storage (low temperatures decrease TAC to 4%–26%) [99].

The antioxidant capacity of raspberries, similarly to other berries, is correlated with various bioactive compounds that have antioxidant properties. Anthocyanins, tannins, total phenolics, and ascorbic acid were studied widely due to their possible correlation. Phenolic compounds, such as *p*-coumaric acid or ellagic acid and their esters, are supposed to be more highly correlated to antioxidant capacity than anthocyanins, than ascorbic acid [96,111,126]. However, the overall antioxidant capacity may be clarified by insight into the connection of different BAC, working additively or synergistically in relation to the total antioxidant capacity of raspberry. Raspberries with higher contents of phytochemicals showed higher antioxidant capacity [112].

## 4. Blackberries

Blackberries (family: Rosaceae, genus *Rubus*, common cultivated variety: *Rubus fruticosus*) have a similar morphology to raspberries; it is an aggregate fruit consisting of many drupelets.

The popularity of blackberries is rising worldwide. They are cultivated mainly in Europe and North America (USA), with similar planted areas. The largest blackberry production regions in Europe are Serbia (90% of their production is processed and exported), and Hungary. Additionally, wild blackberries make a significant contribution to worldwide production (15,000 tons harvested in 2005) [42].

Blackberries are known for curing and preventing a wide variety of ailments, such as colitis, in folk medicine [127]. Blackberries are considered to be a promising sources of active compounds with neuroprotection qualities against age-related diseases, such as neurodegeneration. Digested metabolites from wild blackberries (*R. brigantinus* and *R. vagabundus*), in quantities that could be found in human plasma, could protect neuronal cells against oxidative damage that is an influential attribute of neurodegeneration [128]. Polyphenol extracts from blackberry also possess anti-inflammatory properties [129,130]. Blackberry polyphenol extract strongly inhibits NO production without cytotoxicity and can also inhibit colon tumor cell growth in a concentration-dependent manner in *in vitro* cell culture [131]. To obtain the therapeutic amounts of anthocyanins, Dai *et al.* [131] are trying to develop formulations containing blackberry extract to transfer anthocyanins to tumors in a more suitable way. Oral capsules containing blackberry extract may then be transferred to the colon to release a high local concentration of anthocyanins at a tumor or pre-tumor site.

The best flavor quality of blackberries is at full maturity when their color changes from glossy black to dull black with optimum firmness. The firmness is cultivar-dependent and decreases in the later stages of maturation. Fresh blackberries are only seasonally available. Most of the blackberries are consumed in frozen or thermally-processed forms. In processed products, such as canned products, there are significant amounts of polyphenol antioxidants (anthocyanins) leached out of the berries into the brine (21%–33%) during processing and storage [132].

Compounds, such as BAC, extracted from blackberries could also be used to the production of functional foods [133] to increase their biological value. They may positively impact on human health in the prevention of various illnesses. Additionally, ascorbic acid from blackberries could contribute to the positive effects of these berries. The amount is in the interval of 5–30 mg/100 mg fw [96,97,98,134]. Presumably, it is also affected by the environment, such as growth conditions [90]. The exact contents are similar to contents of raspberries but less than in strawberries and about 2–3-fold to the content in red currants and about 8–9-fold less than in black currants [90].

For functional foods the effective processing of bioactive components could be profitable for future advances to increase the recovery of polyphenolic compounds (such as ellagitannins) from fruits [135]. Continuous pressing and the use of enzymatic pretreatment are suitable for products with higher content of polyphenolic compounds, in particular that of ellagitannins and anthocyanins [136].

### 4.1. BAC in Blackberries

The composition profiles of blackberry polyphenolics are qualitatively similar, yet quantitatively very different [137]. The relevant BAC in blackberries are presented in Table 3.

**Table 3:** Phenolic composition and factors influencing the composition of blackberries

Berry	Major Phenolic Compounds	Factors	References
Blackberry	*Phenolic compounds*		
		Cultivar, genotype	[96,137,138,139,140,141]
		Growing location	[97]
		Cultivation condition (maturation)	[137]
		Processing (juicing, pureeing, canning, freezing)	[132,139,142,143]
		Storage (time, temperature)	[120,132,139,142,143,144]
	*Flavonols and Flavons*		[145,146,147]
	Kaempferol glycosides (Kaempferol-gacetylgalactoside, Kaempferol-glucoside)		
	Quercetin glycosides (Quercetin-3-galactoside, Quercetin-3-glucuronide, Quercetin-3-glucoside, Quercetin-3-rutinoside = rutin, Quercetin-3-rhamnoside)		
	Myricetin glycosides (Myricetin-3-galactoside, Myricetin-3-glucoside)		
	*Anthocyanins*		[137,140,143,145,146,147]
	Cyanidin glycosides (Cyanidin-3-glucoside, Cyanidin-3-rutinoside, Cyanidin-3-arabinoside)		
	Pelargonidin glycosides (Pelargonidin-3-glucoside)		
	Peonidin glycosides (Peonidin-3-glucoside)		
	*Phenolic acids and Hydrolyzable tannins*		[142,143,145,147]
	Ellagic acid and its glycosides		
	Ellagitannins (sanguiin H-6 and lambertianin C)		
	Gallic acid and galloyl esters		
	*p*-coumaric acid and coumaroyl glycosides		

Values are slightly decreasing from underripe to ripe stages. Contents of ellagitannins and ellagic acid derivatives dropped in fully ripe fruit, as did flavonols which decreased to half values compared to the unripe stage. Consequently, values for total phenolic compounds decreased, but only slightly, showing no specific trend [148]. The content of total anthocyanin pigments at different maturity stages is different and the increase is about 2–4-fold from underripe to overripe stages of blackberries with the qualitatively same composition [137]. Contents of major anthocyanin pigments increased about seven-fold in fully-ripe fruit [148].

The amounts of total polyphenols are different due to various factors (Table 2), such as environmental factors, light, temperature, and agronomic practices [96,97,137,146].

Processing of blueberries (canning in syrup, water, pureeing, and juicing) and storage are responsible for the significant losses of procyanidins. The least-retained content was in juices, and the most retained in berries canned in syrup and water [149]. These processing methods had insignificant effects on ellagitannins, but juice processing of berries resulted in total ellagitannin losses of about 70%–82%. This could happen due to ellagitannin-rich seeds being removed in the presscake [142]. Additionally, hot-air drying of blueberries resulted in lower TPC [139]. The ellagitannin amount and composition of frozen berries remain stable during storage. Thermal processes, especially blanching, significantly reduce anthocyanin content in blackberries. The final products show decreased values for the anthocyanins cyanidin-3-glucoside (by 52%) and cyanidin-3-malonyl glucoside (64%). Anthocyanins continue to decline during storage, especially if temperatures are high [143]. Juice processing resulted in the largest losses (~67%) over six months of storage, whereas canned products were the least influenced by processing (17.8% and 10.5% losses) for canned-in-water and canned-in-syrup blended cans, respectively. Thermal processing of purees resulted in 27.4% loss in anthocyanins. In canned products considerable amounts of anthocyanins leached out of the berries into the brine (between 21% and 33%) during processing and storage [132,144].

### 4.2. Antioxidant Capacity of Blackberries

Antioxidant capacity of blackberries is influenced by several factors as listed in Table 3.

The antioxidant capacity of blackberries is affected by concentrations of the extract [150] that could be applicable into functional foods as a natural pigment. Ascorbic acid is not an influential contributor to the antioxidant capacity [37,96,144]. A relevant correlation was observed between total polyphenols and TAC, and/or total anthocyanins. The relationship between radical scavenger activity and total polyphenols is qualified as being closer than that between the radical scavenging activity and total anthocyanins [37,96,144,151]. Therefore, both phenolics and anthocyanins influence antioxidant activity considerably.

## 5. Blueberries

Blueberries, blue colored fruits, belong to the genus *Vaccinium*, family Ericaceae. Rabbit eye blueberries (*Vaccinium ashei*), *Vaccinium angustifolium* Aiton (lowbush blueberry) and *Vaccinium corymbosum* L. (highbush blueberry) are classified as commercially-grown plants. In the last decade blueberries have become more popular due to their well-known health benefits, nutritional value, and excellent sensory evaluation.

Worldwide, the USA ranks first in the production of blueberries, supplying 166,786 tons in 2009. In addition to the USA, Australia and Canada are also dominant in blueberry cultivation and production. South Korea is one of the leaders in Asia, and in China and Turkey blueberries have become an important crop [140,152]. The fruit is also native to Europe.

*Vaccinium* berries are known as a significant source of vitamins and other bioactive substances of pharmaceutical interest. Blueberries are among the richest fruits in ascorbic acid. The content is usually in quite wide intervals, between 10–100 mg/100 g fw [2,153,154,155,156]. The concentration of ascorbic acid decreases during storage depending on the storage conditions, such as oxygen level, temperature, and light. Even after short storage the content decreases; after 10-days of fridge storage it decreased to about of 73% of fresh fruit [157,158].

Blueberries have been reported to have a pharmacological impact against ophthalmologic disorders. They improve blood and oxygen delivery to the eye and scavenge free radicals, which contribute to cataract and macular degeneration [159]. Blueberries containing proanthocyanidins, anthocyanins, and flavonols are beneficial in bone protection, too [160]. Blueberries also exhibit anti-diabetic properties and protection of pancreatic β-cells from glucose-induced oxidative stress [161,162]. Clinical study with volunteers consuming blueberry beverages have demonstrated improved insulin sensitivity in insulin-resistant subjects [163]. Blueberries could also be used for decreasing blood pressure, decreasing of blood cholesterol and, therefore, lowering of cardiovascular risk and atherosclerosis prevention [164,165,166]. Del Bo′ *et al.* [167] referred to the effect of 300 g of blueberries intake on selected markers of oxidative stress and antioxidant protection (endogenous and oxidatively-induced DNA damage) and of vascular function (changes in peripheral arterial tone and plasma nitric oxide levels) in males. Blueberries considerably reduced H_2_O_2_-induced DNA damage after blueberry intake. Blueberry phytochemicals could inhibit growth and metastatic potential of breast and colon cancer cells [168,169]. The synergistic effect of polyphenol compounds and ascorbic acid correlate with inhibition of cancer cell proliferation, inhibit the growth of tumor cells, and induce apoptosis [170,171,172]. It was assessed that pure anthocyanins, such as cyanidin 3-glucoside, delphinidin, as well as peonidin 3-glucoside, suppressed growth of human tumor cells and apoptosis of colon and breast cell lines [173,174].

Short shelf life of berries is a common problem, which limits availability and consumption. Blueberries have quite a quick harvest season. They can be stored only six weeks under controlled atmospheric conditions. Generally, blueberries are sold in fresh, frozen, and processed forms (dried and canned fruits, juices and jams, in beverages, yoghurts) for various food applications. More than 50% of mature blueberries are processed into different products. Processing and preservation methods, such as hot air drying, freezing/thawing, freezing/osmotic pretreatment, and microwave drying [175,176,177,178] are popular techniques for blueberry preserving. At present, modified atmosphere packaging, cold and freezer storage, UV irradiation, and sulfur dioxide fumigation are among the postharvest preservation techniques used to eliminate postharvest deterioration, prolong shelf-life, and maintain the biological nutrition of fresh blueberries [179].

### 5.1. BAC in Blueberries

There are relevant variances in the anthocyanins content, TPC, and TAC between individual blueberry species, as well as between varieties and within other *Vaccinium* species. The relevant BAC in blueberries are presented in Table 4.

**Table 4:** Phenolic composition and factors influencing the composition of blueberries.

Berry	Major Phenolic Compounds	Factors	References
Blueberry	*Phenolic compounds*		
		Cultivar, genotype	[91,125,138,146,156,180,181,182,183,184,185]
		Growing location	[140]
		Cultivation techniques (conventional, organic)	[184]
		Cultivation condition (maturation)	[182,185]
		Processing (juicing, pureeing, canning, freezing, blanching)	[149,175,186,187,188]
		Storage (time, temperature)	[149,153,186]
	*Flavonols*		[123,180,189,190]
	Myricetin glycosides (Myricetin-3-glucoside, Myricetin-3-rhamnoside)		
	Quercetin glycosides (Quercetin-3-galactoside, Quercetin-3-glucoside, Quercetin-3-rutinoside)		
	*Anthocyanins*		[89,123,140,146,155,180,183,186,188,189,190,191,192,193,194]
	Cyanidin glycosides (Cyanidin-3-galactoside, Cyanidin-3-glucoside, Cyanidin-3-arabinoside)		
	Delphinidin glycosides (Delphinidin-3-galactoside, Delphinidin-3-arabinoside, Delphinidin-3-glucoside )		
	Malvidin glycosides (Malvidin-3-galactoside, Malvidin-3-arabinoside, Malvidin-3-glucoside)		
	Petunidin glycosides (Petunidin-3-galactoside, Petunidin-3-arabinoside, Petunidin-3-acetylglucoside)		
	Peonidin glycosides (Peonidin-3-galactoside, Peonidin-3-arabinoside)		

The values of total phenolic quantitative analysis in blueberries are in quite a wide interval; in various studies the contents are upwards of 10-times higher or lower, depending on the method used for analysis [2,140,175,195].

It is generally known that the phenolic content in blueberries is influenced by the degree of maturity at harvest, growing practices, and growing locations [182]. The maturity stages increased anthocyanin content [185], on average by 34% [182]. It is suggested that during blueberry ripening there is phenolic conversion toward anthocyanin synthesis that results in an overall decrease of other phenolic compound content.

Regarding the influence of processing methods on blueberry BAC, a slight increase in total anthocyanin value after some thermal pre-treatment processings was examined. Blanching of blueberries at 85 °C for three minutes resulted in about 7% growth of anthocyanin content [175]. However, the anthocyanin content of thermally-treated blueberries, osmodehydrated, or air dried at 70 °C, decreased by about 30% [187]. The amount of anthocyanins after freeze-drying is also lower probably due to their degradation [155]. It could be concluded that air drying treatment has a negative effect on anthocyanins, while blanching and freezing may increase the extractability of anthocyanins from thermally-treated skins.

The influence of storage conditions on anthocyanin stability for blueberries stored frozen was also investigated and an average of about 59% degradation of the anthocyanins was found after six months of storage. Delphinidin-3-glucoside was the compound showing the greatest degradation (almost 80%), whereas pelargonidin-3-glucoside was the most stable (9% loss) [186].

### 5.2. Antioxidant Capacity of Blueberries

Blueberries are fruits with one of the highest antioxidant capacities. The antioxidant capacity of blueberries is influenced by several factors, as listed in Table 4.

The antioxidant activity of blueberry depends on their phytochemical complex, being mainly represented by anthocyanins, procyanidins, chlorogenic acid, and other flavonoid compounds [187]. It is supposed that the major contributors to their antioxidant activity are mainly anthocyanins, responsible for about 84% of TAC, and not ascorbic acid [123]. Ascorbic acid, which is present in blueberries in a significant amount, was found to contribute to antioxidant capacity only with a small portion up to 10% [196,197].

In contrast with some other berries, antioxidant activity of blueberries is higher in early maturation stages and during initial pigmentation than in the ripe stage. This is related to a high level of hydroxycinnamic acids and flavonols before ripening. The lesser antioxidant capacity of mature blueberries may indicate that anthocyanins have lower antioxidant potential than other phenolic compounds, such as flavonols [182].

Regarding cultivar variance, for rabbiteye blueberries it was hypothesized that they could have higher antioxidant activity than lowbush and highbush varieties. This might be due to their thicker skin having higher concentrations of anthocyanins. The variances in total phenolic content between cultivars and maturity stages are relevant for the obtained changes of the antioxidant activity. The contribution of each individual phenolic compound to the total antioxidant capacity may vary [181].

The effect of blueberry processing on TAC was not observed for blanching (85 °C for three minutes) [175] or drying with osmotic treatment [177]. Freezing of the blueberry fruits increased the antioxidant capacity during the first three months of storage, followed by a reduction up to the end of the six months of storage [186].

## 6. Cranberries

The cultivated cranberry (*Vaccinium macrocarpon* Ait., lowbush cranberry), which is also named the American cranberry, belongs to the Ericaceae family. More than 90% of the total world production is produced in the USA (mainly the northeastern part of North America) and Canada. A smaller amount of cranberry production belongs to Chile [198].

Cranberries product range includes fresh fruits, dried fruits, and products such as juices or food ingredients in cereals, meat and milk products, and sauces.

Cranberries contain a lot of biologically active substances; which came to be thought of as one of the novel functional foods and nutraceuticals. They are known as a good source of vitamins, such as ascorbic acid. Its content in cultivated cranberries is, on average, 10 mg/100 g·dry matter (dm), which is about 21% less than in wild cranberries [199]. In fresh cranberries it was evaluated that the content reached about 134 mg/100 g·dm. This vitamin is present in great amount in cranberry juice, at an amount of 897 mg/L [200]. As for the amount of vitamin C in processed cranberry products, the freeze-drying processes causes a decrease in the content. With an increase of drying temperature, a decrease of ascorbic acid content was observed (from 134 mg/100 g·dm to 64 mg/100 g·dm, on average) [201].

Cranberries exhibit various health benefits. As for the consumption of cranberries (juice and various concentrated products), it was investigated that after a single serving of cranberry juice intake the plasma antioxidant level significantly increased for up to 7 h [202]. For an increase of plasma phenolic content and plasma antioxidant capacity the intake of 500 mL of cranberry juice is satisfactory [203]. Cranberries (juice, concentrated powders, capsule formulations, and tablets) are known that could prevent and treat an occurrence of urinary tract infections. This effect is achieved by proanthocyanidins contained in cranberries that can prevent adhering of *Escherichia coli* to uroepithelial cells in the urinary tract [204,205]. Due to this fact, cranberries could also be used for stomach ulcers [206]. Another potential health effect of cranberries is the finding that extracted compounds from cranberry have shown the prevention and reduction of the cardiovascular disease risks and protection against lipoprotein oxidation [207,208]. It has been demonstrated that the hydroxycinnamic acid derivatives and flavonoids from cranberry juice can reduce not only the oxidation of LDL but also its mobility and, thus, reduce one of the significant critical steps in the atherosclerotic process, which is oxidation of LDL-cholesterol. In addition, cranberry extract could significantly elevate synthesis of hepatic LDL receptors. The synergistic effect of phenolics is then responsible for increasing uptake of cholesterol by hepatocytes [202,209]. In the last decade, *in vitro* anti-cancer activity, anti-mutagenic effects or anti-tumorigenic activity of cranberries has been examined [210,211,212,213]. Some of these biological effects have been generally linked to the incidence of phenolics in cranberries [214].

### 6.1. BAC in Cranberries

Quercetin is the one of the major significant flavonoids occurring in cranberries. Ellagic acid in cranberries represents 51% of the total phenolic compounds in the berries. This constituent occurs in the free form, linked as ellagitannins esterified with glucose or glucosides alone [34]. The relevant BAC in cranberries are presented in Table 5.

**Table 5:** Phenolic composition and factors influencing the composition of cranberries.

Berry	Major Phenolic Compounds	Factors	References
Cranberry	*Phenolic compounds*		
		Cultivar, genotype	[215,216,217]
		Growing season	[218]
		Cultivation condition (maturation)	[185,219]
		Processing (juicing)	[220,221]
		Storage (time)	[216,222]
		
	*Flavonols*		[123,125,223]
	Kaempferol glycosides (Kaempferol-3-glucoside)		
	Quercetin glycosides (Quercetin-3-galactoside, Quercetin-3-arabinoside, Quercetin-3-rhamnoside)		
	*Anthocyanins*		[123,224,225]
	Cyanidin glycosides (Cyanidin-3-glucoside, Cyanidin-3-galactoside, Cyanidin-3-arabinoside)		
	Peonidin glycosides (Peonidin-3-glucoside, Peonidin-3-galactoside, Peonidin-3-arabinoside)		
	Pelargonidin glycosides (Pelargonidin-3-galactoside, Pelargonidin-3-arabinoside)		
	Malvidin glycosides (Malvidin-3-galactoside, Malvidin-3-arabinoside)		
	Delphinidin glycosides (Delphinidin-3-arabinoside)		
	Petunidin glycosides (Petunidin-3-galactoside)		
	*Phenolic acids*		[221]
	*p*-coumaric acid		

As for the total phenolic values during cranberry maturation from green to dark red stages, they decreased in cranberries to half values. Additionally, the amount of monomeric anthocyanins had risen from unripe to ripe stages, more than 100-fold [219].

The conditions during cranberry juice processing (light, oxygen, enzymatic reactions), as well as heating treatment, can influence the stability of the cranberry bioactive compounds. Freezing, in comparison to thermal processing, is better for retention of phenolics, as TPC in frozen cranberries was more than four-fold higher (depending on the type of extraction solution) than in the juice samples (initial pressed juice, clarified juice, and concentrate) [213]. The total phenolic content of cranberries after heat processing slowly decreases, and after 70 minutes of drying is less than 70%; the total anthocyanins were about half of the original ones [201].

### 6.2. Antioxidant Capacity of Cranberries

The great antioxidant properties of cranberries ranks them as one of the best among many other fruits due to phytochemicals, such as benzoic and cinnamic acid derivatives, and flavonols. Antioxidant capacity of cranberries is influenced by several factors as listed in Table 5. Cranberry extracts (processed cranberry juice) differ in their range of phenolic compounds (polar, non-polar, and anthocyanins) and their capacity to scavenge free radicals [220]. TAC of cranberries begins to increase when the cranberries cumulate more anthocyanins [219]. The antioxidant activity in cranberries might increase with the time of drying. The measured increase after drying was about 1.3-times higher than before drying [201].

## 7. Conclusions

Berries, especially members of several families, such as Rosaceae (strawberry, raspberry, blackberry), and Ericaceae (blueberry, cranberry) are great dietary sources of bioactive compounds (BAC). BAC (phenolic compounds such as phenolic acids, flavonoids-flavonols, anthocyanins, tannins, and ascorbic acid) are contained in berries in great amount, and may act as strong antioxidants and, thus, could help in the prevention of inflammation disorders, cardiovascular diseases, or have protective effects to lower the risk of various cancers. The composition and content of BAC in berries is variable depending on the cultivar and variety, growing location, and environmental conditions, plant nutrition, ripeness stage, and time of harvest, as well as subsequent storage conditions or processing methods. This review gives comprehensive information about BAC in each of the selected berries and the factors that influence their antioxidant capacity. The bioactive compounds are of great interest for nutritionists and food technologists due to the opportunity to use BAC as functional food ingredients.
